# Regional Cerebral Blood Flow in *Mania: Assessment Using 320-Slice Computed* Tomography

**DOI:** 10.3389/fpsyt.2018.00296

**Published:** 2018-07-04

**Authors:** Yiming Wang, Xingde Liu, Peifan Li, Haiyan Zhou, Lixia Yang, Lei Zheng, Pingxia Xie, Lingjiang Li, D. Joshua Liao, Qianqian Liu, Deyu Fang

**Affiliations:** ^1^Department of Psychiatry, Affiliated Hospital of Guizhou Medical University, Guiyang, China; ^2^Neuroelectrophysiological Testing Center, Affiliated Hospital of Guizhou Medical University, Guiyang, China; ^3^College Students' Mental Health Education and Counseling Center, Guizhou Medical University, Guiyang, China; ^4^Department of Cardiology, Affiliated Hospital of Guizhou Medical University, Guiyang, China; ^5^Clinical Research Center, Affiliated Hospital of Guizhou Medical University, Guiyang, China; ^6^The Second Xiangya Hospital, Central South University, Changsha, China; ^7^Department of Pathology, Affiliated Hospital of Guizhou Medical University, Guiyang, China; ^8^Department of Pathology, Northwestern University Feinberg School of Medicine, Chicago, IL, United States

**Keywords:** mania bipolar disorder, depression, transcranial doppler ultrasound, 320-slice CT, mitochondria, energy, blood flow

## Abstract

**Objectives:** While evidence that episodes of mania in bipolar I are associated with changes in bioenergetic and regional cerebral blood flow (rCBF) and cerebral blood flow velocity (rCBFV), both the regions and the extent of these changes have not yet been defined. Therefore, we determined the pattern of regional cerebral perfusion mania patients and using patients with major depressive disorder (MDD) as positive controls and healthy participants as negative controls.

**Methods:** Twenty participants with mania, together with 22 MDD patients and 24 healthy volunteers, were recruited for this study. On all participants, Transcranial Doppler (TCD) was conducted to measure rCBFV parameters, 320-slice CT was conducted to measure rCBF in the different cerebral artery regions, and hematological parameters were assessed. ANOVA and Pearson's tests were used for the statistical analysis.

**Results:** Our data indicated that rCBF in the medial temporal lobe and hippocampus, especially in the left medial temporal lobe and the right hippocampus, was increased in the mania group compared with the control and MDD groups (*p* < 0.01). In contrast, rCBF in the medial temporal lobe and hippocampus was decreased in the depression group (*p* < 0.01) compared with healthy controls. In addition, values of rCBFV in the bilateral internal carotid arteries (ICAs) and middle cerebral arteries (MCA) were increased in mania (*p* < 0.01) in comparison to the MDD group. Whole blood viscosity and hematocrit as well as red blood cell sedimentation rate remained unchanged in all group (*p* > 0.05).

**Conclusions:** In mania, rCBF is increased in the medial temporal lobe and hippocampus, with a corresponding increase in rCBFV in the same regions.

## Introduction

Symptomatically, mania in bipolar disorder is characterized by increased energy (1). Conversely depression in bipolar disorder is associated with decreased energy (2). There is evidence showing increased resting energy expenditure in manic episode patients, suggesting an altered regulation of energy and local cerebral blood flow in mania (3). Takeda et al. have observed that the brain advanced function is related to the changes in the cerebral blood flow perfusion, and that both cerebral blood flow and blood flow velocity in certain regions will change when human emotion is changed (4). However, more work is required to better understand the cerebral blood flow involved in bipolar disorder.

*In vivo* neuroimaging studies can assist in understanding the neural regions involved in energy dysfunction in bipolar disorder via analyzing blood flow and metabolic processes. A meta-analysis of comprising 65 functional magnetic resonance imaging (fMRI) studies of 1,074 healthy volunteers and 1,040 bipolar disorder cases, showed abnormal inferior frontal cortex and medial temporal activation in bipolar disorder, especially, in mania, inferior frontal cortex under activation has been observed to relate to both emotional and cognitive processing (5). A other study using positron emission tomography (PET) suggests decreased activity in the anterior cingulated and caudate using positron emission tomography (6). Studies of whole-brain PET imaging examining glucose utilization have shown increases in mania and decreases in depression (7). A recent study with magnetic resonance spectroscopy (MRS) reports that euthymic bipolar disorder subjects fail to replenish ATP from phosphocreatine through creatine kinase (CK) enzyme catalysis during tissue activation in the occipital lobe (8). A novel MRI method sensitive to proton chemical exchange (affected by pH, metabolite concentration, and cellular density) has been used to study subjects with euthymic bipolar disorder vs. controls (9), where consistent metabolic and structural abnormalities in bipolar disorder particularly in cerebral white matter and the cerebellum are discerned. However, other studies using single photon emission computerized tomography (SPECT) report that in comparison to controls, subjects with mania manifest significantly reduced perfusion in many regions, including the left frontal area, left anterior cingulate and parietal cortices (10). Patients with unipolar depression have significantly lowered perfusion than controls in most of the regions examined, mainly in the anterior temporal and frontal cortices bilaterally; they also have lowered perfusion in the right anterior temporal and frontal areas, as well as the right middle temporal area and the right thalamus, compared with manic patients (11).

At present, more and more techniques, including SPECT, positron emission tomography (PECT), TCD, MRI, etc., have been used for cerebral blood flow research. The above-mentioned studies on cerebral blood flow in bipolar disorder are inconsistented, and this methods are just semi-quantitative measurement. The results are just ratio, rather than absolute value of cerebral blood flow. TCD is convenient and easy to do, but it just is a qualitative indicator (12). The PECT image acquisition time is long and difficult to obtain (13). MR perfusion is only used on MR machines with planar echo techniques and cannot be used extensively (14). Since the 320-slice CT was applied to the clinic, whole brain perfusion imaging technology has been successfully applied to the study of cerebrovascular diseases (15). Although both employ the same principle of perfusion imaging, compared with traditional CT, 320-slice CT can extend the original narrow coverage to 160mm, can obtain the whole brain volume data one-time, and can quickly, accurately and stereoscopically measure cerebral blood flow (16). 320-slice CT is more comprehensive for diagnosis and study of cerebrovascular diseases.

In this study, monitoring rCBF using the novel imaging technique our goal was to clarify the pattern of regional cerebral perfusion in cerebral hemispheres of mania with bipolar I disorder that offers quantitative and high-resolution cerebral perfusion analyses, also furthermore in order to clarify its potential utility to psychiatric disorder for possible diagnostic and treatment response purposes.

Specifically, we aimed to compare rCBF in bipolar I disorder to both MDD subjects and healthy individuals as controls to clarify regional cerebral perfusion patterns in the different cerebral areas. The main method employed was the Toshiba Aquilion ONE 320 slice dynamic volume Computed-Tomography (320 slice CT) whole brain one-stop scanning, in combination with Transcranial Doppler (TCD) ultrasound and hematological parameters, to assess rCBF and cerebral blood flow velocity (rCBFV) and blood viscosity. Our novel use of this technique in a study on depression has been outlined recently (17).

## Materials and methods

### Subjects

Twenty patients with the manic phase of bipolar I disorder were selected for the study. They were either hospitalized patients or outpatients with episodes of mania in the Psychiatry Department of Guizhou Medical University Hospital, GuiHang 300 Hospital or the Second People's Hospital of Guizhou Province, from July of 2014 to May of 2016. All examinations were performed before the patients received drug treatment, and all patients did not take antipsychotics for a month before they were hospitalized. All the patients met diagnostic criteria for a manic episode of bipolar I disorder as defined by DSM-IV-TR (Elevated, exaggerated, or irritated mood continues for at least one week, or less, but scratching the extent of hospitalization. At the same time, patients have exaggerated self-evaluation, reduced sleep, more volubility, drifted idea, and so on. These symptoms do not meet the criteria for mixed seizures and are not due to substance or the direct physiological effects, but can result in obvious defects in professional, daily social activities, interpersonal relationships) (diagnosed by two clinicians), a Bech-Rafaelsen Manic Rating scale (BRMS13) >14 (mean, 35.25 ± 10.12), a Hamilton Depression Rating Scale (HAMD24) <8 scores, and a Self-Rating Depression Scale (SDS) <50 scores. The diagnosis criteria for the MDD group (*n* = 22) met with a Hamilton Depression Rating Scale 24 (HAMD24) score of >20 points. A SDS score >53 were used in this study.

Exclusion criteria for all groups included, (i) no history for taking drugs that could influence vessel compliance function upon enrollment (such as stimulants, hypnotics or sedatives) within 6 months, (ii) no other diseases of the nervous system, in particular aneurysms involving the supra-aortic vessels and chronic cerebral venous insufficiency, (iii) no active somatic diseases (e.g., diabetes, hypertension, coronary heart disease, and atherosclerosis) and (iv) no other significant mental disorders.

Twenty-four healthy volunteers, age and gender matched, with a HAMD24 score <20 and without any histories of bipolar I disorder, depression or significant somatic disorders were selected as normal controls. The physical examinations of these healthy controls were performed in the physical examination center of Guizhou Medical University Hospital.

### Ethics statement and consent

The study was approved by the Ethics of Human Investigation Committee of Guizhou Medical University (NO: 20140016) and all procedures were conducted in accordance with relevant guidelines and regulations as well as with the updated Declaration of Helsinki (18). The participants themselves or legally authorized representatives had signed a written informed consent and obtained safeguards in this study.

### Hemorheologic measurement

Venous blood (3 ml) was collected in the morning (08:00–09:00) from each participant, with heparin as an anticoagulant, and used for analyses of blood viscosity (including high middle low shear rates), hemoconcentration, hematocrit (HCT) and red blood cell sedimentation with an instrument named automatic blood rheometer (LBY-N6B, Beijing Precil Instrument Co. Ltd.).

### Transcranial Doppler screening method

Each subject was assessed using the 2 MHz probe transcranial color-coded Doppler (TCD) sonography (Germany, DWL-X type), in accordance with the guideline of Hua-Yang TCD ultrasound practice and with the diagnostic criteria (19), at 9:00 am on an empty stomach in a quiescent condition. The mean flow velocity (Vm), systolic peak velocity (Vs), diastolic velocity (Vd) in the middle cerebral artery (MCA), anterior cerebral artery (ACA), and inferior cerebral artery (ICA) were detected through different bone windows as we described previously (17). The Pulsatility Index (PI) was calculated as PI = (peak systolic velocity end diastolic velocity)/mean blood flow velocity. Data was generated by the TCD analysis software *via* the trace envelope of the measured arterial spectrum and a series of blood flow parameter values.

### Regional cerebral blood flow measurement and perfusion image analysis methods

After collection of clinical and demographic data, subjects were studied using TCD screening. All subjects were measured for rCBF in the different cerebral artery regions using the 320 slice CT (20) (Japan's Toshiba Aquilion ONE, non-helical scan mode, 912-channel, 16 cm coverage, lap rotation time 0.5 s, slice thickness 0.5 mm, vision 240 mm). Assessment was done at 10:00 am on an empty stomach with the method described previously (17). The area of interest was selected on the whole brain perfusion image to measure regional cerebral blood flow (rCBF). These interests are in line with the following requirements: (1) Located between the Reid baseline cross-section and the paracentral lobule cross-section. (2) Located in the frontal lobe, temporal lobe, basal ganglia, and hippocampus. (3) The size of the region of interest is 1 cm2. (4) The blood flow in the area is greatly affected by the emotional state. (5) Avoid large blood vessels. (6) Symmetrical selection. (7) The same position is selected for each patient.

### Statistical analyses

Data were analyzed using SPSS Version 22.0 and presented as means ± SEM. Chi-square of independent samples and one way ANOVA were used to determine the significant difference among groups with *P* < 0.05 considered significant. Dunnett-t was used to multiple comparisons. A series of Pearson's correlations were carried out to determine the strength and the relationship between rCBF and rCBFV parameters, with 95% confidence intervals used. Multiple linear regression was conducted to analyze these factors (including age, mania and depression) that influence rCBF and rCBFV. Simple randomization was conducted using SAS version 9.1.

## Results

### Clinical features

All participants were selected with both age and gender matched. Therefore, there were no significant difference among the bipolar I disorder, MDD and control groups observed for demographic variables in age (mania group: 19–60 years old mean 32.74 ± 14.27 years old; control group: 19–60 years old, mean 42.29 ± 9.54; and depression group: 19–60 years old, mean 39.86 ± 14.25) and other parameters (including sex, blood pressure, smoking, and hypertension), as shown in Table 1. In addition, hematological parameters, including blood viscosity (high middle low shear rate), hematocrit and red blood cell sedimentation, did not show significant differences between the depression and control groups (Table 2).

**Table 1:** Comparisons of demographic variables amongdifferent groups.

**Factors**	**Groups**	**Mania (*n* = 20)**	**Control (*n* = 24)**	**Depression (*n* = 22)**	**F/X2**	***P-*value**
Age		32.74 ± 14.27	42.29 ± 9.54	39.86 ± 14.25	3.156	0.052
Sex	M	8 (40%)	10 (41%)	8 (36.4%)	2.825	0.086
	F	12 (60%)	14 (59%)	14 (63.6%)		
Smoking	Yes	4 (20%)	6 (25%)	5 (22.7%)	1.375	0.548
	No	16 (80%)	18 (75%)	17 (77.3%)		
Alcohol drinking	Yes	17 (85%)	20 (83.3%)	16 (72.7%)	1.394	0.532
	No	3 (15%)	4 (16.7%)	6 (27.3%)		
Hypertension(>140/90 mmHg)	Yes	4 (20%)	5 (20.8%)	7 (31.8%)	1.524	0.439
	No	16 (80%)	19 (79.2%)	15 (68.2%)		

**Table 2:** Comparisons of hematological parameters among the mania, normal and depression groups ($\bar{x}\pm s$).

**Items**	**Mania (*n* = 20)**	**Control (*n* = 24)**	**Depression (*n* = 22)**	***P-*value**
High shear rate (mPa.s/150S 21)	4.44 ± 0.61	4.53 ± 0.45	4.21 ± 0.50	0.900
Middle shear rate (mPa.s/60S 21)	5.32 ± 0.72	5.36 ± 0.58	5.04 ± 0.60	0.925
Low shear rate (mPa.s/10S 21)	9.35 ± 1.92	8.71 ± 1.23	8.32 ± 1.40	0.901
Hematocrit	0.46 ± 0.04	0.45 ± 0.03	0.45 ± 0.03	0.971
Red blood cell sedi-mentation (mm/h)	28.17 ± 11.61	35.39 ± 5.54	34.47 ± 7.35	0.803

### Comparisons of rCBF among the mania, depression, and control groups

**Figure 1:**
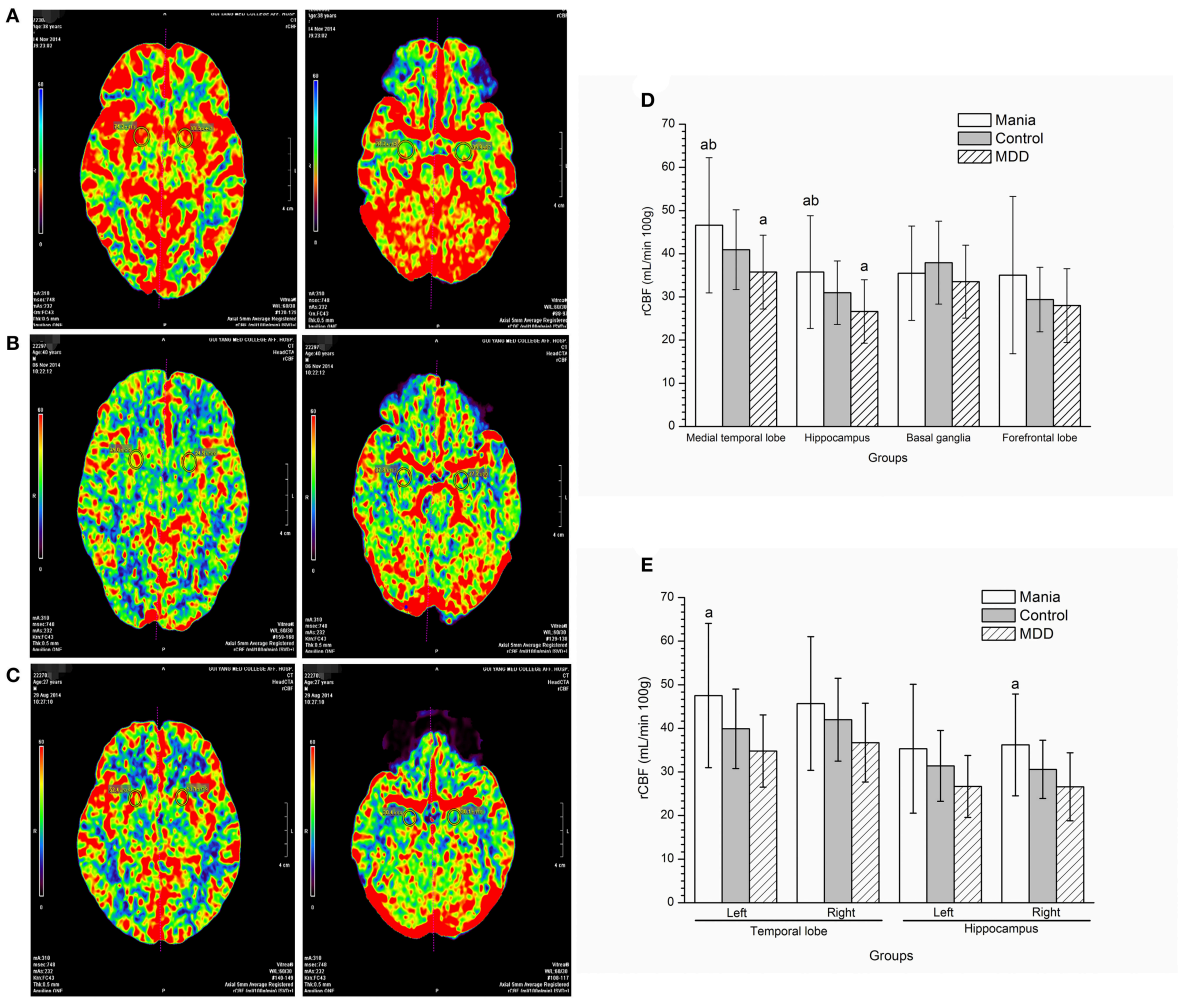
Comparisons of regional cerebral blood flow among the mania, depression and control groups. In the picture, red represents the highest rCBF, yellow represents high rCBF, green represents low rCBF, and blue represents the lowest rCBF. **(A)** Perfusion image of the medial temporal lobe (Left) and hippocampus (Right figure) cerebral blood flow in a mania patient (male, 38 years old). **(B)** Perfusion image of the medial temporal lobe (Left) and hippocampus (Right figure) cerebral blood flow in a MDD patient (male, 40 years old). **(C)** Perfusion image of the medial temporal lobe (Left) and hippocampus (Right figure) cerebral blood flow in a normal participant (male, 37 years old). **(D)** Comparison of regional cerebral blood flow among groups. ^a^*P* < 0.05, compared with the control group, ^b^*P* < 0.01, compared with the MDD group. **(E)** Analyses of regional cerebral blood flow in left and right hemispheres among groups. ^a^*P* < 0.05, compared with the depression group.

Compared with the control and the depression groups, rCBF in the medial temporal lobe and hippocampus was all increased in manic patients (*P* < 0.005). In contrast, rCBF in the medial temporal lobe and hippocampus was reduced in the MDD group compared with the healthy controls (*P* < 0.05) (Figure 1D, photo Figures 1A–C, left: temporal lobe, right: hippocampus). Notably from Figure 1E, rCBF in the left medial temporal lobe and right hippocampus was much higher in the mania group than in the depression group (*P* < 0.05).

### Comparisons of rCBFV among the mania, depression, and control groups

**Figure 2:**
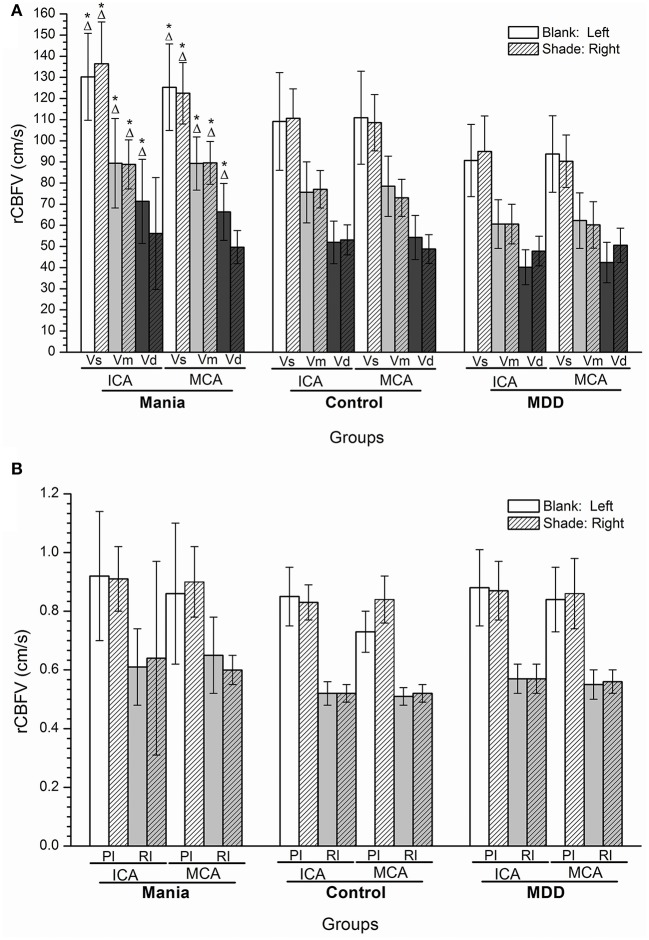
**(A,B)** Histogram of regional cerebral blood flow velocity of disease groups and the control group. *P < 0.05, Compared with the control group; ^Δ^*P* < 0.05, Compared with the depression group. ICA, internal carotid artery; MCA, middle cerebral artery; Vs, systolic velocity; Vd, diastolic velocity; Vm, mean velocity; PI, pulsatility index; RI, resistance index.

Compared with the control and depression groups, the values of Vs, Vd, and Vm of rCBFV in the left ICA and MCA were increased in the mania group, and the Vs and Vm values in the right ICA and MCA (*P* < 0.05). In contrast, the values of Vs, Vd, and Vm of rCBFV in the left ICA and left MCA were decreased in the depression group, and the Vs and Vm values in the right MCA and the right ICA, compared with the control group (*P* < 0.05). However, no significant differences were discerned in values of PI and RI of rCBFV in the ICA and MCA among all three studied groups (Figures 2A,B).

### Relationship between rCBF and rCBFV in mania

**Figure 3:**
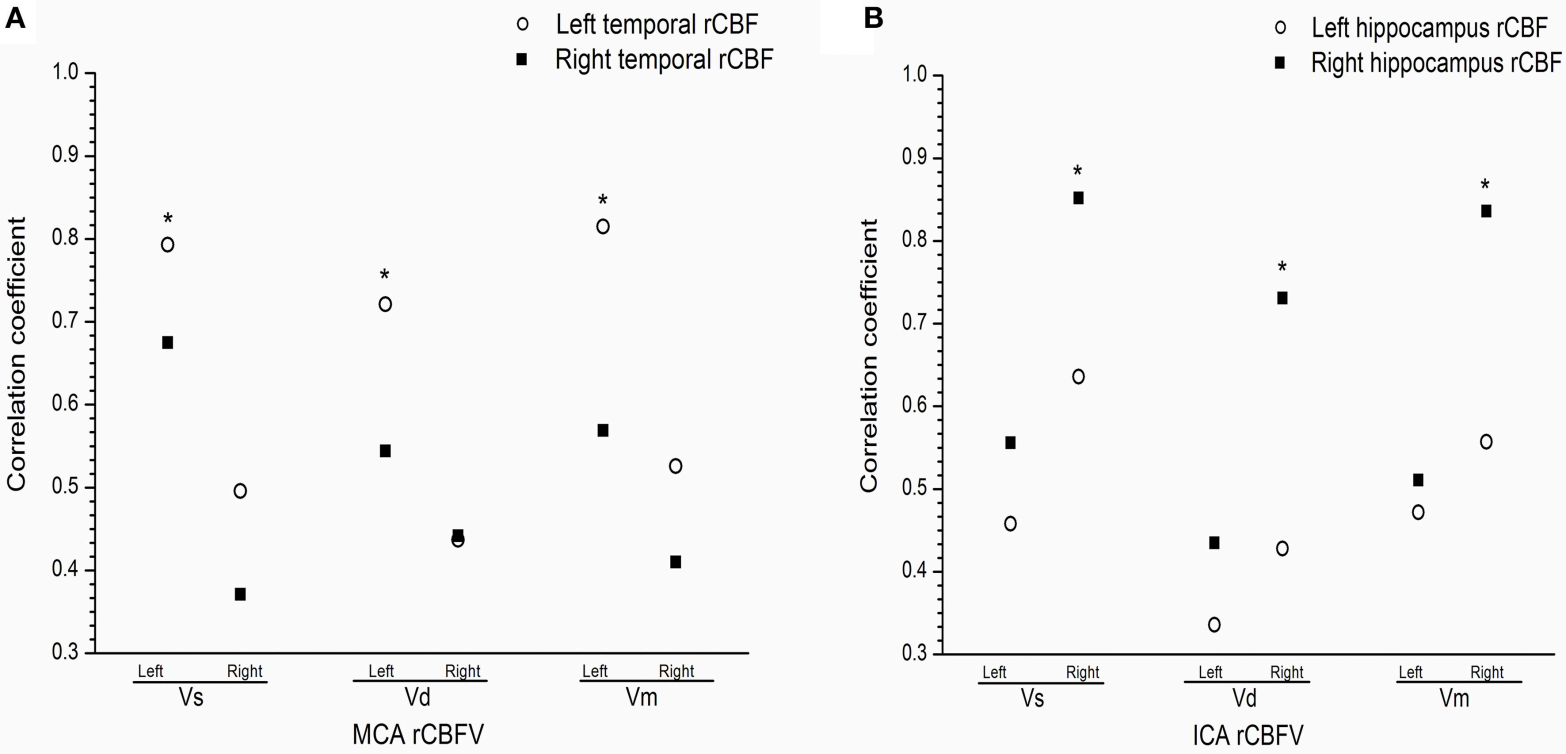
**(A)** Correlation between regional cerebral blood flow and flow velocity in the medial temporal in the mania group. **(B)** Correlation between regional cerebral blood flow and flow velocity the hippocampus in the mania group. Correlation coefficient of *T-*test, *P < 0.05. ICA, internal carotid artery; MCA, middle cerebral artery; L, Left; R, right; Vs, systolic velocity; Vd, diastolic velocity; Vm, mean velocity.

There was a positive correlation between rCBFV and rCBF in the left medial temporal lobe and the right hippocampus in the mania group, such that there was a positive relationship between rCBFV (including MCA-L-Vs, MCA-L-Vd, MCA-L-Vm, MCA-R-Vs, MCA-R-Vd, MCA-R-Vm, ICA-L-Vs, ICA-L-Vd, ICA-L-Vm, ICA-R-Vs, ICA-R-Vd, and ICA-R-Vm) and rCBF in the medial temporal lobe and the hippocampus in the mania group (*r* = 0.815, *P* < 0.05) (Figures 3A,B).

### The effects of mania, depression, and age on rCBF and rCBFV

We found that age and depression had negative effects on rCBF in the medial temporal lobe and hippocampus, and rCBFV in MCA and ICA, while manic had positive effects on rCBF in the medial temporal lobe and hippocampus, and rCBFV in MCA and ICA. In particular, both manic and depression can still affected rCBF in the medial temporal lobe and hippocampus, and rCBFV in MCA and ICA after the exclusion of age (Tables 3, 4) (*R* = 0.376, *P* < 0.05).

**Table 3:** Multiple linear regression analysis for association of covariate with rCBF.

**Factors**	**Medial temporal lobe**			**Hippocampus**		
	**St B**	**T**	***P-*value**	**St B**	**T**	***P-*value**
Mania	0.206	1.785	0.032	0.197	1.546	0.021
Depression	−0.170	−1.993	0.024	−0.155	−2.034	0.018
Age	−0.321	−3.519	0.007	−0.345	−3.322	0.003

**Table 4:** Multiple linear regression analysis for association of covariate with rCBFV.

**Factors**	**ICA(Vs)**			**MCA(Vs)**		
	**St B**	**T**	***P-*value**	**St B**	**T**	***P-*value**
Mania	0.234	1.667	0.018	0.285	2.243	0.005
Depression	−0.166	−1.006	0.015	−0.236	−2.115	0.008
Age	−0.254	−2.214	0.009	−0.245	−2.013	0.009

## Discussion

Using 320 slice CT imaging to measure rCBF and rCBFV in MDD, as articulated in our previous study (17), is novel in psychiatry study and leads to the observations that rCBFV is positively correlated with the corresponding vascular rCBF in both gray and white matters, that prominent changes occur in grey matter blood flow, and that rCBF of the left gray matter is lower than its right counterpart in MDD. In this study, we use this approach for the first time in study of mania in bipolar I and obtain intriguing data.

Available information regarding perfusion and metabolic activity in mania is quite controversial. Some studies show increases in various brain regions (3, 21) while some others show decreases (5, 8–10). Deckersbach observed increased rCBF in the left dorsolateral prefrontal cortex in patients with bipolar disorder, also associated with episodic memory and learning (22). However, another study not only indicated significantly reduced perfusion in the left frontal, anterior cingulate and parietal cortices areas in mania patients but also showed a close correlation between the severity of psychotic symptoms and reduced rCBF (10). Hyper-perfusion of frontal and temporal lobes was detected in patients with bipolar disorder, potentially indicative of over-activation of these areas secondary to emotion modulation (23). Ota M et al. reported that BD patients showed a positive correlation between rCMR (region cerebral metabolism rate) and rCBF in most regions (24). Benabarre et al. (25) found that increased rCBF in cingulate cortex was associated with decreased executive functioning in mania without treatment. In particular, O'Connel et al. found increased rCBF in striatal and temporal regions during the manic phase (26). Another study showed a reduced global CMR in the depressed state compared to controls (27), suggesting functional significance for increased temporal blood flow in mania, with an overlay of significant frontal and temporal lobe gray matter structural findings onto functional findings (28).

We specifically studied the hippocampal function, looking at its metabolism in mania. Roda et al. showed a progressive fall in hippocampal and brain gray matter density in patients with BD (29), while another study found changes in DNA methylation in the human hippocampus in bipolar disorder and schizophrenia (30). Hypothesizing as to the significance of increased blood flow in mania, we need to include this information: in the left hippocampus, there is decreased neuron integrity in mania patients (31), there are decreased hippocampal volume in BD, and we need to overlay the significance of structural findings onto functional findings (32).

In this study, we observed that rCBF in the left medial temporal lobe and right hippocampus was increased in the mania group, compared with the depression group. In Gonul et al.'s report (13), during BD, increased rCMR and rCBF were found in hyperactive subcortical limbic activity (including ventral striatum and amygdala) using PET or SPECT image, neuronal networks were thought to be regulated by serotonin in the limbic system, and abnormal 5-HTT density distribution in BD was relevant to the dysfunction of fronto-limbic network.

In Savitz et al.'s postmortem study in patients with bipolar disorder, reduced amygdala and hippocampus volume were observed as well (33). These structural changes positively correlate with blood oxygenated level-dependent (BOLD) activity or rCBF in response to affective or rewarding reaction after a glutamate-driven excitotoxic process.

Paralleling the above neuroimaging findings, in this study, we observed nascent evidence suggesting abnormalities in cerebral blood flow in mania. Di Tommaso examined perfusion lateralization and found right hyperperfusion and left hypoperfusion during depression, with the converse pattern in mania (34). Luo et al. observed that rCBFV increased in ACA, MCA, posterior cerebral artery, and the vertebral basilar artery in patients with mania (35). Agarwal observed increased CBV in the left frontal and temporal regions in bipolar disorder (23). Although we observe that the whole blood viscosity and hematocrit are not significantly different among different groups, this phenomenon still awaits further confirmation with many more cases.

Results from limited studies using semi-quantitative measurement do not necessarily objectively reflect rCBF changes. In the study, we found that the values of Vs, Vd, and Vm of rCBFV in the left ICA and MCA were increased in mania, and the Vs and Vm values in the right ICA and MCA were similarly increased. We also observed a positive relationship between rCBFV and rCBF in the left medial temporal lobe and the right hippocampus, suggesting increased rCBFV and rCBF in the medial temporal lobe and hippocampus, largely in the left medial temporal lobe and the right hippocampus region. The increase in flow velocity is likely a compensatory mechanism to increase the regional metabolic demands. However, whole blood viscosity (including high middle low shear rate) and hematocrit are not significantly different among the three groups in our study. Of course, the changes of rCBFV and the rCBF in the brain are often affected by complex factors including physical and mental activity, sleep deprivation, temperature change, hydration level, antipsychotics, lithium, etc. In our study, some exclusion criteria were applied for all groups upon enrollment, including drugs that influence vessel compliance function, diseases of the nervous system, somatic diseases, or other significant mental disorders.

The strength of this paper is the novelty of the use of CT methodology. Given the scant literature of its use in psychiatry, more replication studies of its utility would be invaluable. While we used an active depression control group, the use of an active control group consisting of individuals with bipolar disorder in the depressive phase would have greater face validity with regards to our hypothesis. It would be useful to have other measures of cerebral bioenergetics. The sample size, while solid for an imaging study, was not extensive. Being able to contrast these CT findings with a validated measure of blood flow, however, increases the accuracy of these findings such as that these are subjects to unrecognized residual confusion due to demographic or other variables. Key more related clinical covariates need to be considered in the future study. We chose specific cerebral hemodynamic regions in patients with bipolar 1 disorder that were obtained by drawing on various regions of interests, which were perfused by the ICA, MCA, and ACA blood supplies, and then by comparing them with the ROIs of the normal and depression patients. This captures representative rCBF, but not the actual value of the cerebral region flow.

We acknowledge some limitations of this study, ours was a relatively small sample taken from patients of different hospital in Guiyang city and had rCBF assessment, this could result in a possible bias, while changes in rCBF were shown, potential clinical applications need further investigate. Secondly because in recruited adults the hemodynamic specific regions in the brain were obtained by regions of interests encompassing the regions of white and gray matter, and then comparing them with the regions of the normal brain, this captured representative rCBF, there may be little difference compared with the real value of the regions of the brain.

## Conclusions

Our results suggest that rCBF is increased in the medial temporal lobe and hippocampus in mania patients. There are prominent changes in the left medial temporal lobe and the right hippocampus region, accompanied by increased rCBFV in the left MCA and the right internal carotid artery (ICA). It will be interesting to study how the changes observed in this study respond to clinical therapy. We believe that the patients with mania were found out the regional cerebral perfusion pattern, realize more patients accompanying emotional high, thinking active, energetic, along with the land transfer and cognitive dysfunction may be linked to increased blood flow to the brain, cerebral blood flow velocity, the rapid proposed theory support for clinical application. Therefore monitoring rCBF in cerebral hemispheres of mania is in order to clarify its potential utility to psychiatric disorder for possible diagnostic and treatment response purposes; furthermore, the treatment of patients with mania in clinical work may pay more attention to the changes of patients' cerebral blood supply.

## Author contributions

YW and XL wrote the paper. PL, HZ, QL, LY, LZ, and PX performed research. LL and DL designed experiments. DF analyzed data.

### Conflict of interest statement

The authors declare that the research was conducted in the absence of any commercial or financial relationships that could be construed as a potential conflict of interest.
